# Novel insights into epigenetic drivers of oropharyngeal squamous cell carcinoma: role of HPV and lifestyle factors

**DOI:** 10.1186/s13148-017-0424-5

**Published:** 2017-11-28

**Authors:** Paolo Boscolo-Rizzo, Carlo Furlan, Valentina Lupato, Jerry Polesel, Elisabetta Fratta

**Affiliations:** 1 Department of Neurosciences, ENT Clinic and Regional Center for Head and Neck Cancer, Treviso Regional Hospital, University of Padova, Treviso, Italy; 2 Division of Radiotherapy, Centro di Riferimento Oncologico, IRCCS-National Cancer Institute, Aviano, PN Italy; 3 Unit of Otolaryngology, General Hospital “S. Maria degli Angeli”, Pordenone, Italy; 4 Unit of Cancer Epidemiology, Centro di Riferimento Oncologico, IRCCS-National Cancer Institute, Aviano, PN Italy; 5 Immunopathology and Cancer Biomarkers, Centro di Riferimento Oncologico, IRCCS-National Cancer Institute, Aviano, PN Italy

**Keywords:** Oropharyngeal cancer, Epigenetics, Human papillomavirus, Environmental risk factors

## Abstract

In the last years, the explosion of high throughput sequencing technologies has enabled epigenome-wide analyses, allowing a more comprehensive overview of the oropharyngeal squamous cell carcinoma (OPSCC) epigenetic landscape. In this setting, the cellular pathways contributing to the neoplastic phenotype, including cell cycle regulation, cell signaling, DNA repair, and apoptosis have been demonstrated to be potential targets of epigenetic alterations in OPSCC. Of note, it has becoming increasingly clear that HPV infection and OPSCC lifestyle risk factors differently drive the epigenetic machinery in cancer cells. Epigenetic changes, including DNA methylation, histone modifications, and non-coding RNA expression, can be used as powerful and reliable tools for early diagnosis of OPSCC patients and improve prognostication. Since epigenetic changes are dynamic and reversible, epigenetic enzymes may also represent suitable targets for the development of more effective OPSCC therapeutic strategies. Thus, this review will focus on the main known epigenetic modifications that can occur in OPSCC and their exploitation as potential biomarkers and therapeutic targets. Furthermore, we will address epigenetic alterations to OPSCC risk factors, with a particular focus on HPV infection, tobacco exposure, and heavy alcohol consumption.

## Background

Head and neck squamous cell carcinoma (HNSCC) is a frequently lethal cancer that mainly develops in the mucosal epithelial lining of oral cavity, hypopharynx, oropharynx, or larynx. In 2020, HNSCC is expected to affect approximately 833,000 new patients worldwide and 151,000 in Europe, thus representing about 5 and 4% of all new cancers, respectively [1]. Generally, HNSCCs are more common in men than in women and in people aged 60 years than in younger persons [1]. Tobacco use and excessive alcohol intake represent the main risk factors for HNSCC development, and they can act synergistically to increase the risk of this malignancy [2, 3]. To date, oropharyngeal SCC (OPSCC)—which comprises SCC of the base of tongue, tonsillar region, soft palate, and the posterior wall of the pharynx between nasopharynx and the hypopharynx—represents a significantly higher proportion of HNSCC [4]. Among alcohol abstainers, tobacco smokers reported a twofold increased risk of OPSCC compared to never smokers, with a dose-response relationship for both intensity and duration [5]. Similarly, regular alcohol drinking was associated to increased OPSCC risk among never smokers, with risks peaking in people drinking ≥ 5 drink/day [5]. In the last decades, a decrease in the incidence of HNSCC from non-oropharyngeal sites has been observed as the results of preventive strategies to reduce tobacco smoking [6]. Conversely, in economically developed countries, incidence of OPSCC did not show such a decline, despite the reduction of tobacco smoking [7]. This suggests that the control of tobacco smoking epidemic has brought out the burden of OPSCC cases associated with high-risk human papillomaviruses (HPV) infection [8]. HPV-driven OPSCC are rapidly increasing in several Western countries [9], mainly topographically restricted to the oropharynx [10], and exhibit a survival benefit compared to HPV-unrelated tumors [10].

**Fig. 1:**
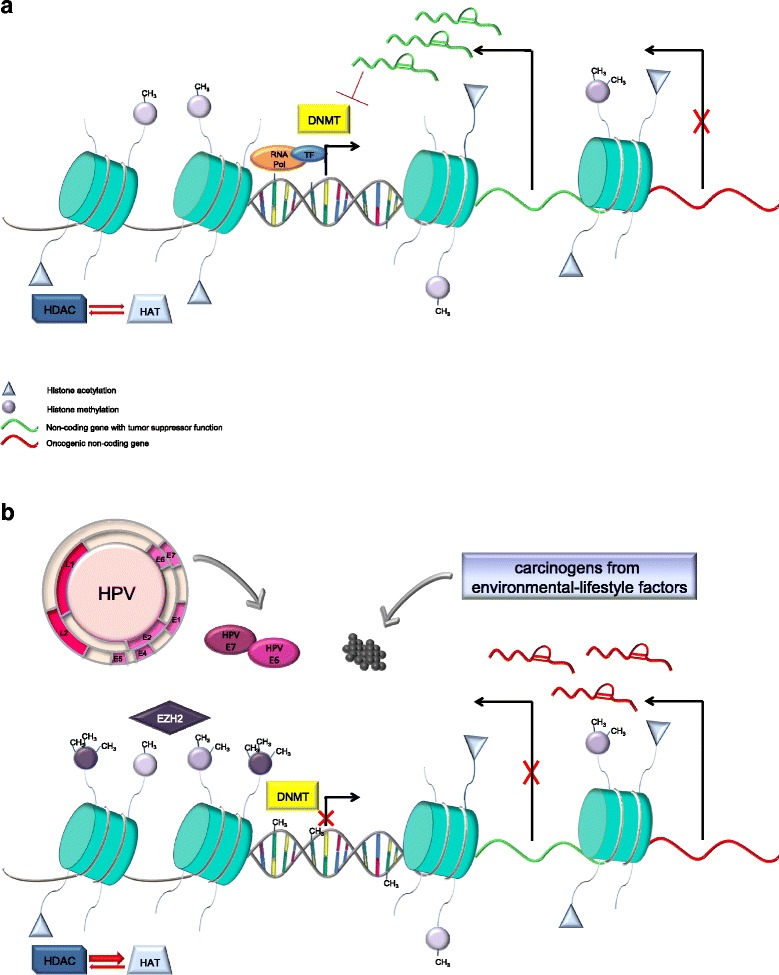
Epigenetic regulation of gene expression involves the crosstalk of DNA methylation, histone modifications, and non-coding RNA (ncRNAs). In normal cells (**a**), CpG within promoter regions of tumor suppressor genes (TSG) are not methylated and are occupied by complexes including RNA polymerase (RNA pol) and transcription factors (TF), thus allowing gene transcription. Histones undergo several post-translational modifications on their N-terminal tails, including acetylation and methylation. Histone acetylation is the result of the dynamic interplay between histone acetyltransferases (HATs) and histone deacetylases (HDACs), and acetylated histones have been associated with actively expressed genes. Unlike histone acetylation, methylation of histone proteins can result in both repressive or promoting effects on transcription, depending on which residue is modified. NcRNAs, which are involved in almost all major cellular functions, may function either as oncogenes or as TSG. NcRNAs whose expression is increased in tumors may be considered as oncogenes, whereas ncRNAs whose expression is decreased in tumor cells are considered TSG. NcRNAs can also regulate the expression and/or the activity of the epigenetic enzymes, such as DNA methyltransferases (DNMTs). In oropharyngeal squamous cell carcinoma (**b**), the epigenetic state of cells changes in response to HPV infection and environmental-lifestyle factors (i.e., tobacco smoke and/or excessive alcohol intake). The result is the accumulation of several aberrant epigenetic modifications that lead to inappropriate activation or inhibition of key signaling pathways. For example, E6 and E7 HPV oncoproteins and carcinogens from cigarettes and alcohol have been demonstrated to affect histone acetylation and methylation patterns either by directly interacting with epigenetic enzymes [i.e., DNMT, enhancer of zeste homolog 2 (EZH2)] or by modulating the ncRNA landscape

Besides genetic alterations, the accumulation of aberrant epigenetic events deeply influence OPSCC biology and may contribute, at least in part, to the differences between HPV-driven and non-HPV-driven OPSCC [11]. To date, the most extensively characterized mediators of epigenetic modifications are DNA methylation and the post-translational modifications of histone proteins [12]. Despite not yet having been extensively characterized, also non-coding RNAs (ncRNAs) are emerging as important factors in the epigenetic determination of gene expression [12–16]. Rather than acting separately, these epigenetic regulators are dynamically connected to each other in the regulation of gene expression. Disruption of this complex epigenetic control mechanism can affect the structure and the integrity of the genome and alter the expression of genes critically involved in tumorigenesis. Of note, it has becoming increasingly clear that environmental and lifestyle risk factors can promote a wide range of epigenetic alterations that are causally involved in cancer development and progression [17]. Based on these considerations, the primary objective of this review is to resume the common epigenetic events in OPSCC and to discuss their potential translational applications for the management of this disease. Any discussion in this review will also relate epigenetic alterations to OPSCC risk factors, with a particular focus on HPV infection, tobacco smoking, and excessive alcohol intake (Fig. 1).

## HPV-driven OPSCC

HPVs are a heterogeneous family consisting of five phylogenetic genera (alpha, beta, gamma, mu, and nu HPVs, encompassing at least 120 genotypes) of small non-enveloped, circular, double-stranded DNA viruses targeting the basal cells of stratified epithelia of the genital and upper respiratory tracts and the skin. Based on their oncogenic potential, the alpha genus (mucosal) HPV types are divided into two groups: low-risk (LR) HPVs, which are mainly associated with benign genital warts, and high-risk (HR) HPVs, which are causative agents of cervical, anogenital, and oropharyngeal cancers (reviewed in [18]). The HPV genome is organized into three regions: a non-coding region, termed the long control region (LCR), regulating gene expression and replication, and two protein-coding regions, the early (E) region coding proteins regulating viral transcription (E2), viral DNA replication (E1, E2), cell proliferation (E5, E6, E7), and viral particle release (E4) and the late (L) region coding for two structural viral capsid proteins (L1 and L2). E5, E6, and E7 are thus viral oncogenes and studies on mucosal HR HPVs have demonstrated that E6 and E7 play a key role in both benign proliferation and malignant transformation, and their continuous expression is critical in maintaining the cancer phenotype in infected cells (reviewed in [19]). In the last two decades, HR alpha HPVs, and in particular HPV type 16 (HPV16), have been causally related to a subset of OPSCC arising from the crypt epithelium of the palatine tonsils and base of tongue [20, 21] as well as to a substantial fraction of SCCs from unknown primary metastatic to the neck nodes [22] to which they confer a more favorable prognosis [23–27]. HPV-driven OPSCC is now considered a rising sexually transmitted disease showing distinctive epidemiological, clinical, and molecular features [4]. Patients with HPV-driven OPSCC are more likely to be younger, without a history of smoking and alcohol abuse, and have a higher socio-economic status and better performance status than those with non-HPV-driven OPSCC [28]. While non-HPV-driven OPSCC show molecular aberrations similar to those observed in SCC of the lung, HPV-driven tumors share similarities with cervical cancer. *TP53* and *CDKN2A/RB1* axes are the most frequently deregulated signaling pathways in both HPV-driven and non-HPV-driven HNSCC [29]. Most environmental-induced cancers harbor inactivating mutations in the *TP53* gene leading to the loss of tumor suppression activity [30]. Furthermore, the p16^INK4a^-cyclin D1-RB axis is mainly deregulated by deletion or promoter hypermethylation of the *CDKN2A* gene encoding p16^INK4a^ [31] and/or by *CCND1* amplification [32], which encodes cyclin D1, with both leading to a decrease in the growth-suppressive hypo-phosphorylated RB form. Conversely from environmental-related HNSCC and consistently with HPV-mediated carcinogenesis, cells from HPV-driven OPSCC rarely contain loss-of-function *TP53* mutations or *CDKN2A* inactivation and show less genomic instability [33]. In this subset of cancers, the p53 and RB pathways are both inactivated as a result of sequestration by binding viral oncoproteins. The E6 protein drives cell proliferation by stimulating ubiquitination and proteasome-dependent degradation of the p53 protein tumor suppressor protein [34]. E7 viral oncoprotein disrupts the RB/E2F complex, resulting in the dissociation of E2F transcription factors from RB-family proteins, thus inducing S-phase entry [35]. Furthermore, viral integration into host genome may contribute to neoplastic transformations by deregulation of key cellular genes and induction of genome instability [36].

## DNA methylation

DNA methylation, catalyzed by DNA methyltransferases (DNMTs), usually occurs at the 5′ position of the cytosine ring within the cytosine-guanine dinucleotides (CpG). Although five members of the DNMT family have been identified, only DNMT1, DNMT3A, and DNMT3B have functional enzymatic activity in mammals. DNMT1 has been called “maintenance” DNMTs since it has a substrate preference for hemi-methylated substrate after DNA replication. Conversely, DNMT3A and DNMT3B are regarded as de novo DNMTs since they create new methylation patterns during embryogenesis and germ-cell development by methylating CpG dinucleotides previously unmethylated on both strands. DNA methylation is associated with repression of genes involved in development and plays a crucial function in genomic imprinting and in X-chromosome inactivation. Besides its role in gene regulation, DNA methylation prevents chromosomal instability by silencing endogenous retroviral and parasitic repetitive sequences (reviewed in [37]). Alterations in DNA methylation patterns have been extensively documented in cancer and appear to deeply contribute to its biology. DNA hypermethylation acts as an alternate and/or complementary mechanism to gene mutation or deletion, resulting in the inactivation of specific gene expression and function of tumor suppressor genes (TSGs) that promote the acquisition of tumorigenic behaviors, such as increased proliferation, enhanced invasiveness, and escape from apoptosis. Besides DNA hypermethylation, the genome of cancer cells undergoes an overall decrease in the level of 5-methylcytosine. This genome-wide hypomethylation affects intergenic and intronic regions of the DNA, particularly repeat sequences and transposable elements, and is believed to facilitate chromosomal instability, loss of imprinting, and reactivation of endogenous parasitic sequences [38].

### Impact of aberrant DNA methylation in HPV-positive and HPV-negative OPSCC

The list of genes that are silenced by DNA methylation in OPSCC is growing rapidly and includes genes involved in several pathways, including apoptosis, cell cycle, DNA repair, and WNT signaling. A selection of the most frequently hypermethylated genes in OPSCC is given in Table 1. Notably, differences in DNA methylation profiles between HPV-positive and HPV-negative OPSCC have been frequently observed in several studies. Overall, while HPV-negative cancers are mainly characterized by genome-wide hypomethylation, the HPV-positive counterpart displays higher levels of promoter methylation (Table 1).

**Table 1:** Genes hypermethylated in OPSCC

Pathway	Gene	Name	Hypermethylated in	Region analyzed	Reference
Apoptosis	DAPK	Death-associated protein kinase 1	HPV-negative/positive	Promoter region	[39]
	RASSF1	Ras association domain-containing protein 1	HPV-negative	− 244 from TSS^a^	[50]
				Promoter region	[45, 51]
	STAT5	Signal transducer and activator of transcription 5	HPV-negative	+ 42 from TSS	[50]
Cell cycle	CCNA1	Cyclin A1	HPV-positive	Promoter region	[51]
				+ 7 from TSS	[50]
	CDKN2A	Cyclin-dependent kinase inhibitor-2A	HPV-negative	Promoter region	[43–45, 51]
			HPV-positive	Three loci within the CpG island of CDKN2A gene	[165]
	CHFR^b^	Checkpoint with forkhead and ring finger domains	HPV-negative	Promoter region	[39]
	TP73	Tumor protein p73	HPV-negative/positive	Promoter region	[39]
Cell fate determination	APC	Adenomatous polyposis coli	HPV-negative/positive	Promoter region	[39]
DNA repair	MGMT	O6-methylguanine-DNA methyltransferases	HPV-negative	− 272 from TSS	[50, 51]
Protein glycosylation	TUSC3	Tumor suppressor candidate 3	HPV-positive	+ 29 from TSS	[50]
Inflammation	JAK3	Janus kinase 3	HPV-positive	+ 64 from TSS	[50]
Invasion and metastasis	CADM1	Cell Adhesion Molecule 1	HPV-positive	Promoter region	[39]
	CDH11	Cadherin 11	HPV-positive	− 354 from TSS	[50]
	CDH13^a^	Cadherin 13	HPV-negative/positive	Promoter region	[39]
	IGSF4	Immunoglobulin superfamily member 4	HPV-positive	Promoter region	[166]
	SPDEF	SAM pointed domain-containing Ets transcription factor	HPV-negative	+ 116 from TSS	[50]
	TIMP3	TIMP metallopeptidase inhibitor 3	HPV-positive	Promoter region	[39, 51]
	SYBL1	Synaptobrevin-like 1	HPV-positive	− 349 from TSS	[50]
Signaling	ESR1	Estrogen receptor 2	HPV-negative/positive	Promoter region	[39]
	ESR2	Estrogen receptor 2	HPV-negative	+ 66 from TSS	[50]
	GALR1	Galanin receptor type 1/2	HPV-positive	Two loci within the CpG island of the GALR1 gene	[165]
	GRB7	Growth factor receptor-bound protein 7	HPV-positive	− 160 from TSS	[50]
	RARβ^a^	Retinoic acid receptor β	HPV-negative/positive	Promoter region	[39]
Transcription	RUNX1T1	RUNX1 translocation partner 1	HPV-positive	+ 145 from TSS	[50]
	TCF21	Transcription factor 21	HPV-positive	Promoter region	[167]
WNT signaling	SFRP1	Soluble frizzled receptor protein 1	Drinkers	Promoter region	[56]
	SFRP4	Soluble frizzled receptor protein 4	HPV-positive	Promoter region	[56]
	WIF1	WNT inhibitory factor 1	NA	Promoter region	[54]

*NA* not applicable

^a^ *TSS* transcription start site

^b^Hypermethylation of these genes is associated with development of radioresistance in other tumor types

#### Apoptosis

Defects in the apoptotic pathways are essential for cancer development and progression, but also for resistance to chemotherapy and radiotherapy. Thus, identification of genes related to apoptosis in OPSCC may offer newer therapeutic modalities. The pro-apoptotic gene death-associated protein kinase (DAPK) is commonly hypermethylated in at least 20% of OPSCC independent of HPV status, indicating it is involved in both HPV-positive and HPV-negative OPSCC carcinogenesis [39]. DAPK gene encodes for a calcium/calmodulin-regulated serine/threonine kinase that is required for apoptosis induced by interferon-gamma [40].

#### Cell cycle

Cell cycle regulation depends on the appropriate expression of cyclin-dependent kinases (CDKs), their binding partners, and the inhibitory molecules such as cyclin-dependent kinase inhibitor-2A (CDKN2A). By using different first exons, *CDKN2A* encodes two overlapping, but very disparate proteins, p16^INK4a^ and p14^ARF^, which are both involved in negatively regulating cell cycle progression through the pRB and the p53 pathways, respectively [12]. The *CDKN2A* locus, which is frequently hypermethylated in tumors, is often overexpressed in HPV-positive OPSCC [41]. Consistently, immunohistochemical staining of p16^INK4A^ protein is commonly used as a surrogate marker for HPV infection in OPSCC [42]. To date, any correlation was observed between HPV status and *CDKN2A* promoter methylation [43–45], thus suggesting that all of the *CDKN2A* promoters of HPV-positive tumors may not be methylated. However, Schlecht et al. have recently identified four hypermethylated *CDKN2A* loci downstream of the p16^INK4A^ and p14^ARF^ transcription start sites. Interestingly, the hypermethylation of this region was associated with p16^INK4A^ protein expression and correlated with an increased expression of p14^ARF^ in OPSCC. Although the involvement of HPV proteins in the methylation of the CpG loci downstream of the CDKN2A gene promoter remains unclear, this may represent a potential mechanism for p16^INK4A^ overexpression in HPV-positive OPSCC [46].

#### DNA repair

The maintenance of cellular integrity depends on the efficiency of multiple specific DNA repair pathways (reviewed in [47]) which are crucial in protecting against genomic instability, a characteristic of tumor development [48]. Genotoxic exposure to carcinogens such as tobacco often results in DNA damage that represents an important mechanism of OPSCC etiology [49]. Aberrant DNA methylation affecting the O6-methylguanine-DNA methyltransferases (*MGMT*) gene has been revealed in HPV-negative OPSCC [50, 51], but also in lung cancer [52], suggesting the loss of the repair function of MGMT may reduce the ability of cells to repair DNA damage in smoking-induced tumors.

#### WNT signaling

WNT proteins belong to a large family of secreted glycoproteins activating several pathways, including the best-known Wnt/β-catenin or canonical pathway [53]. Aberrant methylation of the negative regulators of this pathway has been observed in HNSCC, including OPSCC [54]. Downregulation of the soluble frizzled receptor protein (*SFRP*) genes, encoding soluble antagonists of WNT protein receptors, has been described as an alternative mechanism of stabilization and activation of β-catenin [55]. The promoter methylation status of these genes was examined in 350 patients with HNSCC, of which 25% derived from the oropharynx. Of note, *SFRP1* aberrant methylation occurred at a higher prevalence in both heavy and light drinkers, whereas *SFRP4* promoter methylation was detected more frequently in never and former smokers and was also associated with HPV16 infection [56].

#### Radiotherapy resistance

Some genes, which are associated with the development of tumor radioresistance, are frequently hypermethylated in OPSCC [11, 57] (Table 1). However, the molecular mechanism by which their inactivation may contribute to radiotherapy resistance in OPSCC is yet to be determined. Among these, checkpoint with forkhead and RING finger domains protein (CHFR) was hypermethylated in 25% of HPV-negative OPSCC patients, while no promoter methylation of this gene was observed in HPV-positive group [11, 57]. *CHFR* silencing was associated to the upregulation of *PARP1*, a gene coding for a DNA repair enzyme involved in radiotherapy resistance in HNSCC [58, 59].

In contrast to gene-specific hypermethylation, which usually occurs in HPV-positive OPSCC, genome-wide and global hypomethylation are more frequently observed in HPV-negative tumors [60], likely leading to chromosomal instability. Although the exact mechanism of global genomic DNA hypomethylation in HNSCC has not fully elucidated yet, differences in the expression and/or activity of DNMTs may explain HPV-related differences among OPSCC [46, 61]. Consistently, methylation levels of the long interspersed nucleotide element-1 (LINE-1) repetitive elements, a widely accepted surrogate of overall genomic DNA methylation content, were shown to be higher in HPV16-positive than in negative HNSCC [60, 62]. This finding suggests that HPV16-infected cells may attempt to silence the virus by DNA methylation, which can result in increased methylation of LINE-1 repetitive elements [62].

### Methylation of HPV genome in HPV-associated OPSCC

HPV genome harbors CpG dinucleotides within conserved palindromic sequences [63]; thus, its DNA strands may be potentially targeted by covalent alterations such as methylation. As HPV genome does not encode any proteins with methyltransferase activity, the methylation of HPV-DNA is supposed to be under the control of the human host cell DNMT. It was shown that HPV-E7 is capable to edit DNA methylation by forming a complex with DNMT1 [46, 64]. In addition, both DNMT1 [65] and DNMT3a [61] were found to be more highly expressed in cells from HPV-positive HNSCC than in those from HPV-negative tumors.

Overall, the analysis of the methylation pattern of the integrate HPV16 and human host genome in cultured cells from HNSCC, including SCC from the base of the tongue, revealed that the methylation status of HPV16 is dramatically affected by the methylation status of the host DNA flanking the integration site with HPV16-DNA being highly methylated when integrated into intergenic highly methylated host genome sites, while remaining largely unmethylated when incorporated into poorly methylated intergenic regions [66]. This observation would argue for a bystander role of the virus methylome rather than an important phenomenon in HPV-driven carcinogenesis.

Different changes of the HPV methylome were observed in relation to squamous epithelial differentiation suggesting that HPV-DNA methylation is a more dynamic phenomenon both in the context of the viral life cycle and progression towards transforming infection modes [67]. The HPV genome includes a non-coding LCR, or upstream regulatory region, which contains a large number of cis-responsive elements governing HPV gene expression and replication. The p97 promoter at the E6 proximal part of the LCR regulates the transcription of E6 and E7 viral oncogenes [68]. HPV16 polymorphisms in the LCR may alter the oncogenic potential of the virus by enhancing p97 promoter activity [69]. Furthermore, numerous mutations were uncovered in the LCRs from oral cancer cells and HPV-immortalized oral epithelial cells which increase the expression of HPV-transforming proteins [70]. Therefore, the methylation status of LCR was intensively investigated both in cervical cancer and OPSCC. The functional significance of CpG methylation in the LCR may be indeed an attempt by the host cell to silence the expression of viral genes or a virus-induced strategy to shift from the productive stage of the viral life cycle towards the transforming phase of HPV infection [67]. Unexpectedly, two research groups observed an unmethylated status of the CpG sites within LCR in the majority of OPSCC samples with integrated HPV16 genome [71, 72]. On the other hand, consistently with previous findings derived from cervical cancer, they found a CpG methylation enrichment at the boundary of the L1 and L2 viral gene. In cervical cancer, the rate of hypermethylation at the L1 and L2 sites was observed to rise progressively with the increasing severity of the lesions [73], and HPV16 CpG methylation at L1 and L2 sites was a reliable biomarker of pre-cancer that can potentially stratify the risk in HPV-positive women [74]. Regardless of the oncological consequences of L1/L2 methylation which remains unknown, the authors suggest its potential utility as diagnostic biomarker of HPV-driven OPSCC [72].

The level of viral oncogene E6/E7 expression is regulated by binding of E2 to E2-binding sites (E2BSs). In particular, at high concentration, E2 binds low-affinity E2BS3 and E2BS4 resulting in inhibition of the p97 promoter and thereby maintaining low levels of E6 and E7 (reviewed in [75]). Thus, the transition towards a transforming HPV infection requires the inactivation of E2. The main mechanism by which this is achieved is the linearization of the viral DNA within the E2 open reading frame [76]. However, in about 60% of OPSCC HPV16 integration appears not necessary for viral transformation [36, 77]. In addition, viral integration in host genome may result in head-to-tail concatemers of full-length HPV16 genomes [78]. In the above two contexts, other mechanisms may contribute to the inhibition of E2 functions. Interestingly, tumor samples from OPSCC patients harboring intact E2 sequences (episomal state or integrated head-to-tail concatemers) displayed intermediated to complete methylation of E2BS3 and E2BS4 and high methylation levels at these sites were closely associated with the highest E6 and E7 expression levels and worse prognosis [79, 80]. Methylation status of E2BSs was critical in maintaining the transformed phenotype in oral SCC cells, as demethylation of HPV16 LCR by 5-aza-2′-deoxycytidine (5-AZA-CdR) caused repression of E6 and E7 expression followed by cell cycle arrest at G2/M [80]. Thus, in the presence of an intact E2 open reading frame, methylation at E2BSs in the LCR of E6 and E7 appears to positively regulate their expression. On the contrary, in a context of E2 gene disruption, the selective pressure on cellular clones harboring methylated LCR site is lost.

### Impact of lifestyle risk factors in OPSCC aberrant DNA methylation

Tobacco smoke has been associated with both TSG promoter hypermethylation and genome-wide hypomethylation, especially in long-term tobacco users, along with prolonged alcohol consumption [81–83]. Tobacco and its metabolites influence the methylation profiles in cancers by impairing DNMT1 and DNMT3 expression, both at transcript and protein level, and by altering its enzymatic activity (reviewed in [84]). The genotoxic exposure to cigarette smoke condensate and heavy metals that are present in tobacco smoke has been also associated with global DNA hypomethylation [85]. Cigarette smoke decreases acid folic and vitamin B_12_ levels [86], which are required for the maintenance of methylation patterns in DNA [87]. Consistently, a reduced concentration of folate was found in the buccal mucosal cells of tobacco smokers [88]. Although these evidences indicated that deficiencies of folate and vitamin B_12_ may be associated with increased risk of developing OPSCC, several CpG sites were found to be differentially methylated in HPV-negative OPSCC patients with the highest levels of both vitamin A and vitamin B_12_ intake. Interestingly, vitamin B_12_ intake was positively correlated with *RARβ* hypomethylation in HPV-positive OPSCC patients, thus emphasizing the differences in tumor biology between HPV-positive and HPV-negative OPSCC [89]. Excessive alcohol intake has been reported to affect DNA methylation by altering folate metabolism and transmethylation reactions (for reviews see [90, 91]). Alcohol can also reduce or increase DNMT activity [92, 93], consistent with the evidence that within the nucleus accumbens core chronic low-level drinking promoted DNA hypomethylation, whereas high levels of drinking resulted in CpG hypermethylation [94]. Despite the limited number of studies that have looked for associations between DNA methylation and alcohol consumption, an analysis on cancers of the upper aerodigestive tract revealed increased levels of *CDKN2A* promoter methylation among alcohol drinkers [95].

## Histone modifications

Histone (H) 2A, H2B, H3, and H4 core histones represent abundant nuclear proteins involved in the chromatin architecture since they enter into the constitution of nucleosomes. Core histones display N-terminal tails that protrude from the nucleosome and are subject to combinations of covalent modifications including acetylation, methylation, phosphorylation, sumoylation, and ubiquitination. These modifications determine how tightly the chromatin is compacted, playing a decisive role in modulating gene expression, as well as serve as docking stations for protein recognition modules which recruit specific functional complexes (reviewed in [96]). Histone acetylation and methylation are most commonly associated with carcinogenesis [37].

Histone acetylation is the result of the dynamic interplay between histone acetyltransferases (HATs) and histone deacetylases (HDACs). In general, transcriptional activators recruit HATs, which alter nucleosomal conformation to produce an open chromatin structure where transcription factors and co-activators can bind to turn on gene transcription, whereas transcriptional repressor associates with HDACs, which promote a more condensed and inactive chromatin state. HATs and HDACs target not only histone tails, but also non-chromatin proteins [97]. Histone methylation, catalyzed by histone methyltransferases (HMTs), occurs at both arginine and lysine residues on the tails of histone proteins H3 and H4. Similar to acetylation/deacetylation, histone methylation is reversible, and demethylation is catalyzed by histone demethylases (HDMs). Unlike histone acetylation, methylation of histone proteins can result in either activation or repression, depending on which residue is affected. Indeed, trimethylation of histone H3 lysine 9 (H3K9), 27 (H3K27), and histone H4 lysine 20 (H4K20) is associated with silent chromatin and transcriptionally inactive genes. Conversely, methylation of lysines 4, 36, and 79 on histone H3 (H3K4, H3K36, and H3K79) can experience various methylated states, including monomethylated, dimethylated, and trimethylated which is closely linked with active transcription [98, 99]. Myeloid mixed-lineage leukemia (MLL) is a HMT that targets several lysine residues on histones, including H3K4, and usually acts as a positive transcriptional regulator. A whole-exome sequencing analysis identified inactivating mutations in MLL2 and MLL3 genes in both HPV-positive and HPV-negative OPSCC [100], indicating a tumor suppressor function. In the same study, the mutational spectrum of HPV-negative tumors resulted very similar to those observed in lung and esophageal squamous cell carcinomas and included mutations of the HMT nuclear-receptor-binding SET-domain-containing 1 (NSD1), which preferentially targets H3K36 methylation [100]. By analyzing public genomic and epigenomic data sets from HNSCC, Papillon-Cavanagh et al. have recently identified a DNA methylation cluster that exclusively contained samples carrying NSD1 mutations or H3K36 alterations. Results were further validated in an independent cohort of OPSCC samples, in which the presence of H3K36 alterations associated to a drastic decrease in H3K36 methylation levels [101]. Altogether, these findings suggest that NSD1 mutations and/or H3K36 alterations may be associated with a genome-wide hypomethylation phenotype in OPSCC.

With the exception of H4, all “canonical” histone proteins in mammals have several variants with different sequences [102]. The “canonical” histones are expressed at high levels during the S-phase of the cell cycle, whereas replication-independent histone “variants” are expressed and incorporated into chromatin throughout the cell cycle (for a review see [103]). Among histone “variants,” phosphorylated H2A.X (γH2A.X) variant represents a useful marker of DNA integrity and repair, because of its ability in recruiting DNA repair proteins at the site of the dysplastic tissue. In response to double-strand breaks (DSB), the serine/threonine kinase ataxia-telangiectasia mutated is activated and rapidly phosphorylates the histone variant H2A.X on S139, forming γH2A.X [104]. Upon DSB induction, γH2A.X appears as subnuclear foci within minutes. The release of γH2A.X foci and the subsequent repair of damage DNA depend on γH2A.X acetylation and subsequent ubiquitination. [105]. Residual γH2A.X foci which are detectable 24 h after damage likely indicate misrepaired or incompletely repaired DSB [106]. Interestingly, Park et al. reported that HPV E7 oncoprotein was able to increase retention of γH2AX nuclear foci following radiation-induced DNA damage. Consequently, the normal kinetics of DNA damage repair was impaired in HPV-positive OPSCC cells both in vitro and in vivo [25]. These findings may explain why HPV-positive tumors are more sensitive to radiotherapy.

### HPV infection and histone modifications in OPSCC

E6 and E7 HR-HPV oncoproteins have been demonstrated to affect histone acetylation and methylation pattern by interacting with HATs, HDACs, HMTs, and HDMs (reviewed in [107]), thus affecting the chromatin landscape of cancer cells. In HPV-driven cancers, histone modifications on targeted genes can mediate bidirectional effects on gene transcription. The interaction between E6 and E7 HPV oncoproteins and the HAT p300/CBP is, in fact, mainly direct towards non-histone targets and aimed to enhance the deregulation of *TP53* and *CDKN2A/RB1* pathways. Independent of its ability to induce p53 degradation, E6 inhibits p300-mediated p53 acetylation, leading to repression of p53-targeted gene activation [108]. By recruiting p300/CBP and pRb, E7 brings the histone acetyltransferase domain of p300/CBP into proximity to pRb and promotes its acetylation, leading to cell cycle deregulation [109]. The C-terminal zinc-binding domain of E7 interacts with HDAC1 and 2 through Mi2β protein, a component of the nucleosome remodeling deacetylase complex, thus inhibiting histone deacetylase activity [110, 111]. Of interest, human keratinocyte-expressing HPV16 E7 show an increased histone H3K9 acetylation on E2F-responsive promoters, which depend on E7 binding with both pRb and HDAC. In addition, methylation of H3K4, which is associated with transcriptional activation, was also increased [112]. This results in the weakening of the histone-DNA interaction at E2F-responsive sites and may promote the transcription of cell cycle progression genes. Enhancer of zeste homolog 2 (EZH2), the functional enzymatic component of the polycomb repressive complex 2 (PRC2), is a HMT catalyzing the addition of methyl groups to H3K27 and, eventually, contributing to formation of a repressive chromatin state [113]. Thus, PRC2 marks transcriptionally silenced genes. Polycomb silencers mediate the repression of key tumor-suppressor pathways and play a crucial role in suppressing genes required for differentiation and maintaining a cancer stem cell phenotype (reviewed in [114]). In vitro studies demonstrated that EZH2 promoter can be activated by HPV E7 oncoprotein via the release of E2F factors from growth-inhibitory pocket proteins [115]. HPV-positive OPSCC have genome-wide elevation in the repressive H3K27me3 histone modification [116], thus confirming HPV-driven carcinogenesis and EZH2 overexpression are closely related. Furthermore, double immunofluorescence quantification of histone lysine methylation revealed that p16-positive OPSCC had global elevation of H3K27me3 and H4K20me1 that are both involved in generating a repressive gene environment through the formation of facultative heterochromatin [117]. Intriguingly, in HPV-positive HNSCC, several PRC2 target genes were found to undergo hypermethylation including members of the cadherin superfamily whose deregulation is implicated in several tumor progression and metastasis processes such as epithelial to mesenchymal transition [118]. Furthermore, a significant enrichment of highly methylated promoter regions of PRC2 targets together with a higher expression of *DNMT3a* was observed in cell lines from HPV-positive OPSCC compared to HPV-negative ones [61]. In contrast to the abovementioned studies, HPV E7 oncoprotein was shown to induce the expression of the lysine demethylases KDM6A and KDM6B causing epigenetic reprogramming mainly by removing the repressive H3K27me3 marks [119]. While in environmental-related carcinomas the cyclin-dependent kinase inhibitor p16^INK4a^ is usually downregulated mainly by gene mutation or deletion, it is frequently overexpressed in HPV-driven tumor and thus, it is considered a surrogate marker for active HPV involvement in OPSCC carcinogenesis. p16^INK4a^ upregulation was considered the effect of transcriptional activation by E2F transcription factor released after E7-mediated disruption of pRb/E2F complexes. But detailed mechanistic investigations suggested that p16^INK4a^ is overexpressed upon HPV E7 oncoprotein signaling via induction of the demethylase KDM6B that removes repressive H3K27me3 marks from the p16^INK4a^ -encoding *CDKN2A* promoter region [119, 120].

### Tobacco smoke may induce chromatin histone modifications in HPV-negative OPSCC

To date, the effect of excessive alcohol exposure and tobacco consumption on histone modifications has not been investigated in OPSCC. However, cigarette smoking was shown to induce specific post-translational modifications in H3 and H4 lysine and arginine during the pathogenesis of smoking-related diseases [121]. In lung cancer, mutations and deregulations of histone-modifying enzymes have been described in association with tobacco smoke condensate [101, 122], and smoke-induced modifications in histone patterns have linked to aberrant gene expression in immune cells [123]. Given that HPV-negative OPSCC most closely resemble lung SCC [124], deregulation of histone-modifying enzymes and chromatin structure may also play a role in tobacco smoke-induced OPSCC.

## NcRNA involvement in OPSCC

NcRNAs are of increasing biologic and therapeutic relevance due to their role in modulating gene expression [12–16]. Generally, ncRNAs less than 200 bp are known as small ncRNAs (sncRNAs) and included small interfering RNAs, micro RNAs (miRNAs), and PIWI-interacting RNAs (piRNAs), while all larger transcripts are defined as long non-coding RNAs (lncRNAs) [125]. NcRNAs can interact with histone-modifying complexes and/or DNMTs, being also targets of these epigenetic mediators [12, 16]. At present, the list of ncRNAs involved in OPSCC includes a large number of sncRNAs (mainly miRNAs) and few lncRNAs (Table 2).

**Table 2:** NcRNAs altered in OPSCC

MiRNA	Up-/downregulation or polymorphism	HPV-associated	Environmental factors associated	Epigenetic regulation	References
Let-7c	Down	No			[127]
MiR-9	Down	No		Promoter methylation	[131]
	Up	Yes			[168, 169, 140]
MiR-18a	Down	Yes			[168]
	Up	No			[127]
MiR-20a	Up	No			[127]
MiR-20b	Up	Yes			[169, 140]
MiR-21	Up	No			[127, 151]
MiR-26b	Down	Yes			[142]
MiR-30a	Up	No	Alcohol		[150]
MiR-30d	Up	No			[127]
MiR-31	Down	Yes			[168]
MiR-93	Up	Yes			[140]
MiR-101	Down	Yes			[142]
	Up	No	Alcohol		[150]
MiR-103	Up	No			[170]
MiR-106b	Up	Yes			[140]
MiR-125a	Down	Yes			[142]
MiR-126	Down	Yes			[140, 142]
MiR-127	Down	Yes			[142]
MiR-130a	Up	No			[127]
MiR-137	Down	No		Promoter methylation	[171]
MiR-143	Down	Yes			[140, 142]
MiR-145	Down	Yes			[140, 142]
MiR-146	Polymorphism	Yes			[129]
MiR-149	Polymorphism	Yes			[129]
MiR-155	Up	No			[170]
		Yes			[168]
MiR-181b/d	Up	No			[170]
MiR-191	Up	No			[170]
MiR-195	Up	Yes			[142]
MiR-196	Polymorphism	Yes			[129]
MiR-198	Down	No			[127]
MiR-199a/b	Down	Yes			[140, 142]
MiR-200c	Up	No			[127]
MiR-222	Up	Yes			[140]
MiR-223	Down	Yes			[168]
MiR-320	Up	Yes			[140]
MiR-363	Up	Yes			[140, 142]
MiR-372	Up	No			[127]
MiR-375	Up		Alcohol		[151]
MiR-379	Down	Yes			[142]
MiR-381	Down	Yes			[142]
MiR-409	Down	Yes			[142]
	Up	No			[127]
MiR-432	Down	Yes			[142]
MiR-433	Down	Yes			[142]
MiR-499	Up	No			[127]
	Polymorphism	Yes			[129]
MiR-517	Down	Yes			[142]
MiR-675	Up	No	Alcohol		[150]
MiR-934	Up	No	Alcohol		[150]
MiR-1201	Down	Yes			[142]
MiR-1266	Up	No	Alcohol		[150]
MiR-3164	Up	No	Alcohol		[150]
MiR-3178	Up	No	Alcohol		[150]
MiR-3690	Up	No	Alcohol		[150]
LncRNA	Up-/downregulation	HPV-associated	Environmental factors associated	Epigenetic regulation	References
CDKN2B-AS	Up	Yes			[135]
EGOT	Up	Yes			[135]
LINC00152	Down	Yes			[135]
NCRNA00185	Down	Yes			[135]
PRINS	Up	Yes			[135]
TTTY14	Up	Yes			[135]
TTTY15	Up	Yes			[135]
XIST	Up	Yes			[135]

MiRNAs represent important mediators of epigenetic regulation of gene expression. MiRNAs can direct endonucleolytic cleavage of the targeted mRNAs or inhibit translation through perfect or nearly perfect complementarity to targeted mRNAs at the 3′ untranslated [12]. To date, limited information is available regarding mechanisms by which miRNA alterations may contribute to OPSCC carcinogenesis and progression. MiRNAs are transcribed in the nucleus by RNA polymerase II into long primary transcripts, which are further processed by two RNase-III enzymes, Drosha and Dicer [126]. Of note, increased expression levels of Drosha, Dicer, and other components of the miRNAs machinery were detected in tonsil SCC [127]. Consistent with these data, a number of miRNAs were upregulated, with the exception of miR-198 and let-7 [127], which has been previously shown to negatively regulate Dicer expression [128]. Among upregulated miRNAs, miR-21 and miR-499 were found to suppress the programmed cell death protein 4 (PDCD4), a tumor suppressor protein that is lost in the majority of tonsil SCC [127]. Apart from miRNA machinery, single nucleotide polymorphisms in miRNA precursors may influence their maturation, and thereby modulate their expression as reported for miR-146, miR-149, miR-196, and miR-499. Polymorphisms in the immature form of these miRNAs were found to significantly increase the risk of HPV16-associated OPSCC, particularly in never smokers [129].

Interestingly, miRNAs have been reported to be epigenetically silenced in laryngeal SCC, oral cavity SCC (OSCC), and OPSCC. In particular, treatment of HNSCC cell lines with 5-AZA-CdR has proven to be effective in restoring the expression of miR-9, one of the best characterized miRNA regulated by DNA methylation in cancer [130]. Furthermore, miR-9 ectopic expression led to PTEN upregulation, and a significant repression of HNSCC proliferation [131]. Thus, miR-9 could represent an important negative regulator of OPSCC cells growth. Other than being regulated by epigenetic mechanisms, miRNAs can in turn modulate the expression of the epigenetic enzymes. Along this line, miR-874 silencing by aberrant CpG promoter methylation has been frequently described in laryngeal SCC, OSCC, and OPSCC. Performing an in silico database analysis, miR-874 was found to negatively regulate the expression of *HDAC1*. Accordingly, luciferase reporter assay demonstrated that miR-874 directly regulated *HDAC1* in HNSCC cells, thus creating a complicated network of reciprocal interconnections [132].

As described above, lncRNAs are defined as RNA transcripts longer than 200 nucleotides that lack protein-coding potential. After transcription via RNA polymerase, lncRNAs are processed are subject to 5′-capping, polyadenylation, and intron splicing. Most lncRNAs are retained in the nucleus, but in some cases, they can also be exported to the cytoplasm. LncRNAs represent potent cis- and trans-regulators of gene transcription and act as scaffolds for chromatin-modifying complexes (reviewed in [133]). At present, the knowledge of lncRNA involvement in carcinogenesis is still in its infancy, largely due to the novelty of these molecules. The putative role of lncRNAs in OPSCC further confirm this, as only few lncRNAs have been studied in detail, and have been directly linked to HPV oncogenic protein activity [134, 135].

### NcRNA deregulation in HPV-positive OPSCC

Although the involvement of ncRNAs in HNSCC is well recognized, only few studies have focused specifically on ncRNAs profile in HPV-positive OPSCC (Table 2) and investigated how HPV modulates their expression [136, 137], since both E6 and E7 HPV oncoproteins were shown to modulate the ncRNA landscape in cancer cells [138, 139]. Using different cohorts of OPSCC, a miRNA panel that differentiates HPV-positive OPSCC from HPV-negative tumors has been recently identified [140]. Interestingly, strong upregulation of miR-9 has been observed in HPV-positive OPSCC but not in HPV-negative tumors [140], in which miR-9 expression was found silenced by promoter methylation [131]. Consistent with this data, increased miR-9 expression associated with HPV activity has been reported in cervical cancer [141]. However, the mechanism by which HPV promotes miR-9 expression is yet to be discovered. In a study of Lajer et al., HPV-positive OPSCC showed a considerable upregulation of miR-363 expression [142], consistent with another report of Wald et al., in which HPV16 E6 knockdown was accompanied by a reduction of miR-363 levels in HNSCC [143], thus suggesting a possible role for miR-363 in HPV-positive OPSCC. MiRNAs can also directly target HPV E6/E7 mRNA, as it has been demonstrated via the ectopic expression of miR-375 mimic in OPSCC and cervical cancer cell lines [144]. Notably, Liu et al. has recently showed that E6 oncoprotein promoted miR-375 epigenetic silencing through overexpression of DNMT1 in HPV16 positive cervical cancer cells [145]. Furthermore, miR-375 was found to negatively regulate the metastasis-associated lung adenocarcinoma transcript 1 (MALAT1) lncRNA [145], which is frequently overexpressed in cancers, and has extensively involved in oncogenic processes [146]. In a previous study of Jang et al., MALAT1 expression increased in oral keratinocytes transfected with HPV16 E6 and HPV E6/E7 oncoproteins, indicating HPV16 may promote cell proliferation by promoting MALAT1 upregulation [134]. A recent study performed on the extensive data set available on The Cancer Genome Atlas portal showed ncRNAs as a significantly upregulated transcriptional RNA population in HPV16-positive HNSCC with the most prominent differentially expressed ncRNAs between HPV16-positive and HPV-negative being associated with protein-coding “targets” involved in the cell cycle and cell-cell signaling [147]. Altogether, these findings suggest the role of ncRNAs in HPV-driven OPSCC warrants future investigation.

### Environmental regulation of ncRNAs in OPSCC

NcRNA expression changes following exposure to environmental carcinogens have been documented in HNSCC. Unfortunately, results were obtained using heterogeneous tumor tissues or cell lines from different sites of HNSCC, so they are not exclusively specific for OPSCC. Overexpression of miR-23a was described in HNSCC from areca-nut chewing patients and correlated with an increase of the DNA damage marker γH2A.X and a reduction of DBS repair [148]. A recent study reported that the downregulation of miR-145 in oral fibroblasts exposed to cigarette smoke condensates promoted pro-tumorigenic stromal-epithelial interactions [149]. A number of aberrantly expressed miRNAs were identified in HNSCC patients, who were subdivided into non-drinkers, light, and heavy drinkers. Among these, miR-30a and miR-934 were the most highly upregulated in drinkers, and their overexpression was associated to the induction of the anti-apoptotic gene *BCL-2* and to increased levels of cell proliferation [150]. MiR-375 was also demonstrated to increase with alcohol consumption in OPSCC [151], but the pathway involved between this miRNA and the excessive alcohol use has not yet been elucidated.

## Epigenetic alterations as diagnostic biomarkers in OPSCC

Epigenetic alterations share some characteristics that render them particularly attractive as clinically applicable biomarkers, since they are characterized by high stability in biologic samples, and they can be easily detected in body fluids (e.g., serum, plasma, saliva, urine). Furthermore, the possibility to amplify them in a cost-effective manner represents a further advantage for routine analyses [152]. The increasingly recognized role of aberrant epigenetic modifications in OPSCC biology strongly suggests for the opportunity to test epigenetic markers as potential indicators of disease prognosis and response to therapy. The ability to determine epigenetic alterations in premalignant lesions, serum, and saliva may also provide valuable biomarkers for the early detection of OPSCC and for monitoring its recurrence.

Consistent with the increasing role of aberrant DNA methylation in HNSCC biology, different studies have reported the methylation of single genes/loci to have a potential in predicting OPSCC clinical outcome. However, a number of them have been conducted on study populations consisted of OPSCC and other HNSCC (Table 3). Taioli et al. studied the methylation of *CDKN2A*, *MGMT*, and *RASSF1* in correlation with OS and tumor recurrence in OSCC and OPSCC. Results demonstrated that *MGMT* promoter methylation was significantly associated with poorer outcome, consistent with the critical role of *MGMT* in DNA repair [45]. In the last years, a number of studies have sought to establish a correlation between promoter methylation and improved survival rate in HPV-positive OPSCC. Along this line, Gubanova et al. provided the evidence that the downregulation of the serine/threonine-protein kinase SMG-1 by promoter hypermethylation correlated with HPV-positive status and improved OPSCC patient survival, and also with enhanced response to radiotherapy in HPV-positive HNSCC cell lines [153]. Subsequently, Kostareli et al. described an HPV-related promoter methylation signature of five genes (ALDH1A2, GATA4, GFR4, IRX4, and OSR2) with strong correlation and predictive power for clinical outcome of OPSCC patients [154]. A more recent study has investigated the influence of the overall level of genomic DNA methylation on early OPSCC relapse risk. Results reported that OPSCC smokers who relapsed within 24 months exhibited significantly reduced methylation levels of the LINE-1 repetitive elements. Interestingly, the association between smoking habits and LINE-1 hypomethylation was stronger in HPV16-negative OPSCC cases [60].

**Table 3:** Association of aberrant DNA methylation and clinical outcome in OPSCC patients

Gene	Hyper-/hypomethylation	Cohort	Clinical outcome	References
MGMT	Hyper	88 OPSCC	Poorer PFS and OS	[45]
ALDH3A1	Hyper	76 HNSCC, including 15 OPSCC	Decreased OS	[172]
TAP1	Hyper			
SMG1	Hypo	40 OPSCC	Improved OS in HPV-positive OPSCC	[153]
ALDH1A2	Hypo	170 OPSCC (3 independent cohorts)	Improved clinical outcome in HPV-positive OPSCC	[154]
GATA4	Hyper			
GRIA4	Hyper			
IRX4	Hyper			
OSR2	Hypo			
DAPK1	Hyper	70 HNSCC, including 9 OPSCC	Lymph node metastasis	[173]
MGMT	Hyper			
WIF1	Hyper	43 HNSCC, including 19 OPSCC	Decreased OS	[54]
GALR1/2	Hyper	202 HNSCC, including 58 OPSCC	Poor survival with the highest association in HPV-negative OPSCC	[174]
LINE-1	Hypo	110 OPSCC (2 independent cohorts)	Increased risk of early relapse in HPV-negative OPSCC	[60]

*ALDH* aldehyde dehydrogenase, *DAPK* death-associated protein kinase, *GALR* galanin receptor, *GATA* GATA binding protein, *GRIA* glutamate receptor, *IRX* iroquois homeobox, *LINE* long interspersed nuclear element, *MGMT* O-6-methylguanine-DNA methyltransferases, *OSR* odd-skipped-related, *SMG* nonsense mediated mRNA decay associated PI3K-related kinase, *TAP* transporter associated with antigen processing, *WIFI* WNT inhibitory factor

Due to their stability as well as the potential role of their dysregulation at different stages of carcinogenesis, ncRNA may represent very promising non-invasive biomarkers for OPSCC. Based on their size and greater stability with respect to mRNA, miRNA represent an attractive target for salivary-based diagnostic [155]. Consistently, several groups have already published miRNA profiles correlated to clinical outcome of OPSCC patients (Table 4). In most cases, the causal relationship has not been completely elucidated. Since the expression of several miRNA is specifically modulated in HPV-driven tumors, miRNA profiles may also be useful for identifying HPV-positive OPSCC patients in the early stage of the disease. The clinical importance of another class of small ncRNA, called piRNAs, has emerged from a study of Firmino et al. in which piRNA expression was assessed in 498 non-malignant and HNSCC tissues, including OPSCC. Data obtained revealed 87 piRNAs that were exclusively expressed in HNSCC, with 41 piRNA clearly associated to HPV status. Among these, 11 piRNAs were significantly downregulated in HPV16/18 tumors compared to other HPV types. Based on these data, authors defined an expression signature of five piRNAs that correlated with OS exclusively in HPV-positive patients, indicating the potential utility of piRNAs in assessing HNSCC patient outcome [156] (Table 4).

**Table 4:** Association of ncRNAs profiles and clinical outcome in OPSCC patients

NcRNA	Conclusion	Cohort	References
MiR-221	MiR-21 expression correlated with poor prognosis in HNSCC patients	147 HNSCC, including 31 OPSCC	[151]
Let-7d	Reduced expression of let-7d and miR-205 is a significant predictor of HNSCC progression independent of anatomical site, tumor stage, treatment, or HPV	104 HNSCC, including 32 OPSCC	[175]
MiR-205			
MiR-107	Associated with overall survival (OS), independent of HPV status	88 OPSCC	[169]
MiR-151			
MiR-492			
MiR-20b	Associated with disease-free survival, independent of HPV status		
MiR-107			
MiR-151			
MiR-182			
MiR-361			
MiR-151	Associated with distant metastasis, independent of HPV status		
MiR-152			
MiR-324-5p			
MiR-361			
MiR-492			
MiR-9	Associated with OS in HPV-positive patients	150 OPSCC	[168]
MiR-18a			
MiR-31			
MiR-155			
MiR-223			
MiR-146	Single nucleotide polymorphisms in these miRNAs were associated with reduced and increased risk of OPSCC recurrence, respectively	1008 OPSCC	[176]
MiR-196			
MiR-193b-3p	Positively associated with OS	81 OPSCC obtained from “The Cancer Genome Atlas”, and 95 OPSCC patients included for validation	[177]
MiR-455-5p			
MiR-92a-3p	Negatively associated with OS		
MiR-497-5p			
PiR-30506	Associated with OS in HPV-positive patients	498 non-malignant and HNSCC tissues, including 66 OPSCC	[156]
PiR-35953			
PiR-36715			
PiR-36984			
PiR-39592			

## Epigenome-modifying enzymes as potential therapeutic targets in OPSCC

Enzymes that maintain and modify the epigenome seem to play a crucial role in OPSCC. In this context, epigenetic drugs might represent an important therapeutic modality for the clinical management of OPSCC patients.

Being regulated by multiple oncogenic pathways and affecting OPSCC phenotype, DNMTs constitute a potential anti-cancer target. So far, the most widely studied DNMT inhibitors (DNMTi) 5-azacytidine (azacitidine, Vidaza) and 5-AZA-CdR (Decitabine, Dacogen) have undergone intensive clinical development that led to their Food and Drug Administration (FDA) approval for patients affected by hematological malignancies [37]. As described above, treatment with 5-AZA-CdR has proven to be effective in restoring the expression of miRNA with tumor suppressor function in HNSCC [131], but also in HPV-transformed cell lines [157]. Considering that HPV-positive OPSCC have been found to have higher levels of TSG promoter methylation, DNMTi might represent an additional treatment option for these patients. Furthermore, an immunomodulatory activity of 5-AZA-CdR, which may ensure efficient therapeutic anti-tumor effects in HPV-positive malignancies, has been also shown in mice vaccinated with HPV DNA vaccines. In fact, 5-AZA-CdR co-delivered with a DNA vaccine encoding calreticulin (CRT) linked to E7 antigen (CRT/E7) was able to increase CRT/E7 DNA expression and to enhance E7-specific CD8+ T cell immune responses [158].

Although the biological consequences of HDAC deregulation in OPSCC are still largely unknown, in vitro studies have reported that HDAC inhibitors (HDACi) could induce cell cycle arrest [159] and promote apoptosis in HNSCC cell lines [160]. Epigenetic drugs have also been explored in combination with chemotherapeutics, indicating they may sensitize HNSCC cells to chemotherapy-induced apoptosis. For example, the combinations of 5-AZA-CdR or HDACi with cisplatin enhanced the cytotoxic effectiveness of this well-established chemotherapeutic in HNSCC treatment [161, 162]. DNMTi also demonstrated some synergistic effect with radiation by reducing HNSCC cell survival compared to the single treatments and by increasing radiation-induced apoptosis [163]. Furthermore, in vivo HDACi administration promoted DNA repair and survival in normal cells after radiation, indicating that HDACi could protect normal tissue from radiation-induced side effects [164]. Based on these promising pre-clinical data, OPSCC clinical trials involving HDACi have been carried out or are on-going (NCT01064921, NCT01695122, NCT01249443, available at www.clinicaltrials.gov).

Unlike DNMTi and HDACi, only a few HMT inhibitors are currently known, and most of them were discovered through random screening approaches [37]. In this context, epigenetic inhibitors targeting the EZH2 pathway have recently shown effectiveness in suppressing OPSCC growth and survival, with a major effect in HPV-positive cell lines [116]. Despite these promising results, no significant decrease in EZH2 and its substrate H3K27 was observed [116], thus indicating the mechanisms of these HMTi need to be further elucidated in OPSCC.

## Conclusions

The incidence of OPSCC is rising rapidly with about 60% of patients presenting with loco-regionally advanced disease at diagnosis and requiring combined modality treatment strategies. Thus, improving survival rate and reducing treatment morbidity are both pressing issues. Epigenetic alterations, including DNA methylation, histone modification, and ncRNAs, clearly impact on key pathways that are involved in OPSCC biology. Epigenetic events occurring in OPSCC should be considered as the consequence of a network of interactions between epigenetic enzymes, on one side, and HPV infection and environmental-lifestyle factors on the other. HPV-positive and HPV-negative OPSCC have singular epigenetic drivers which may impact on different clinical behaviors and treatment response and strengthen the concept that HPV-driven OPSCC are biologically distinct from non-HPV-driven tumors. Expanding our understanding on how epigenetic modifications contribute to OPSCC and enlightening the convergent crosstalk existing between DNA methylation, histone modification, and ncRNA networks may improve the knowledge of its pathogenesis and provide new novel biomarkers for diagnosis or prediction of disease outcome and/or response to therapy. Furthermore, the development of next-generation epigenetic drugs may offer the tools necessary for promising therapeutic treatment of both HPV-positive and HPV-negative OPSCC patients.

## Abbreviations

5-AZA-CdR: 5-aza-2′-deoxycytidine
CDKs: Cyclin-dependent kinases
CHFR: Forkhead and RING finger domains protein
CpG: Cytosine-guanine dinucleotides
DAPK: Death-associated protein kinase
DNMTs: DNA methyltransferases
DSB: Double-strand breaks
E: Early
E2BS: E2-binding site
EZH2: Enhancer of zeste homolog 2
H: Histone
HAT: Histone acetyltransferase
HDAC: Histone deacetylases
HDACi: HDAC inhibitors
HDM: Histone demethylase
HMT: Histone methyltransferase
HNSCC: Head and neck squamous cell carcinoma
HPV: Human papillomavirus
HPV16: HPV type 16
HR: High risk
K: Lysine
L: Late
LCR: Long control region
LINE-1: Long interspersed nucleotide element-1
lncRNA: Long non-coding RNA
LR: Low-risk
MALAT1: Metastasis-associated lung adenocarcinoma transcript 1
MGMT: O6-Methylguanine-DNA methyltransferases
miRNA: Micro RNA
MLL: Myeloid mixed-lineage leukemia
ncRNA: Non-coding RNA
NSD1: Nuclear-receptor-binding SET-domain-containing 1
OPSCC: Oropharyngeal squamous cell carcinoma
OSCC: Oral cavity squamous cell carcinoma
PDCD4: Programmed cell death protein 4
piRNA: PIWI-interacting RNA
PRC2: Polycomb repressive complex 2
SFRP: Soluble frizzled receptor protein
sncRNA: Small non-coding RNA
TSG: Tumor suppressor gene
